# Effects of odors on sleep quality in 139 healthy participants

**DOI:** 10.1038/s41598-022-21371-5

**Published:** 2022-10-13

**Authors:** Agnieszka Sabiniewicz, Pia Zimmermann, Guliz Akin Ozturk, Jonathan Warr, Thomas Hummel

**Affiliations:** 1 Interdisciplinary Center “Smell & Taste”, Department of Otorhinolaryngology, TU Dresden, Dresden, Germany; 2 Dokuz Eylul University, Izmir, Turkey; 3 Takasago Europe Perfumery Laboratory, Paris, France

**Keywords:** Human behaviour, Psychology, Health care

## Abstract

The present study aimed to systematically examine whether laurinal, orange odor, and a specifically designed “perfume” influence sleep quality. During sleep, healthy participants (n = 139) were presented with odor or no odor through nose clips for fourteen consecutive nights (phase one). We collected physiological parameters together with subjective reports. Later on, longer lasting effects of this manipulation were examined for the following fourteen nights (phase two) without exposition to odors. Additionally, olfactory, cognitive and non-cognitive measures were conducted before phase one, between both phases and after phase two. One-way analyses of variance for repeated measures with nights and condition (1 vs 2) as the within-subject factor and odor condition (0, 1, 2 or 3) together with odor pleasantness rating as between-subject factor, was employed to analyse data. Overall, the present results demonstrated that the odor condition in comparison to control had no consistent effect on sleep in healthy participants which can be possibly explained by exposure to odors via nose clips. However, the analyses indicated that the individual pleasantness of odors enhanced the positive assessment of sleep quality. Altogether, the present results indicate that the subjective perception of an odor’s hedonic value appears to be crucial for sleep quality, not the odors themselves.

## Introduction

Fragranced substances, including single or multiple odorants adding up to perfumes or aromas, have been assigned for millennia with a unique role for human mental and physical health^1^. In ancient Egypt, myrrh incense was used to decrease fear and increase sleep quality^2^. More recently, citrus fragrances have been assigned with mood-enhancing properties^3^. A number of scientific reports confirmed the common knowledge and beliefs about the connection between exposure to odors and sleep quality: odors were found to modify neural processes during sleep^4^ and impact dreams and sleep quality^4–7^.

Some odors were attributed with particular effectiveness in improving both objective and subjective sleep quality. Bitter orange odor increased sleep quality in postmenopausal women^8^ and improved mothers’ sleep quality in postpartum period^9^. Jasmine odor led to greater sleep efficiency and reduced sleep movement^10^. Lavender oil, in turn, enhanced sleep effectiveness, increased total sleep time^11,12^, promoted sleep in patients with insomnia^13^ and those having clinical interventions^14,15^.

Scents targeting sleep and well-being, such as lavender, induce central relaxant and sedative effects^16,17^. Generally, plant-derived odors can enhance mood and calmness and are typically assessed as pleasant^18^. Pleasant odors per se positively affect mood and decrease arousal^19–21^. Pleasantness of odorants is also related to higher molecular complexity^22^. Recently, Ackerley et al.^23^ demonstrated that two pleasant odors activated olfactory-relevant areas and showed some beneficial impact on sleep.

Given the common anecdotal and subjective evidence in aromatherapy research, systematic objective investigations on the impact of odors on sleep quality are few^11,24^. Pleasant odors were found to have some beneficial effects on sleep quality in healthy humans^8,13,23,25^. However, other studies did not confirm this effect^6,26,27^ and Nováková et al.^28^ indicated no impact of odors on sleep quality as measured by dream content. Overall, the effects of odors on sleep quality are relatively superficially studied and still require a more profound understanding.

Hence, the purpose of the present study was to systematically examine whether l-Laurinal® ([S]3,7-dimethyl-7-hydroxyoctanal), orange odor (essential oil), and a specifically designed, complex perfume (further termed as “perfume”; provided by Takasago, Paris, France), influence sleep quality of a large group of healthy participants. We aimed to investigate the difference between particular odors in causing this effect. Specifically, we intended to investigate the effects of odor exposure during sleep on physiological parameters and subjective reports.

## Materials and methods

### Participants

A total of 192 participants were enrolled in the study, of whom 53 were excluded due to various reasons: pregnancy or breast-feeding; significant health problems possibly related to olfactory disorders (e.g., Parkinson's disease, renal insufficiency); acute or severe chronic inflammation of the nose or nasal cavities; heavy smoking (more than five cigarettes a week); rough night sleep times; any allergic reaction to the silicon.

Thus, the final sample consisted of 139 participants (82 women, 57 men; mean age 29 years, SD = 9.6, range 18–66 years of age). Data were collected at the Smell & Taste Clinic at the Department of Otorhinolaryngology of the TU Dresden. The study was performed according to the principles of the Declaration of Helsinki on biomedical research involving human subjects. The study design was approved by the Ethics Committee at the Medical Faculty of the TU Dresden (EK number 377082019). All participants provided written informed consent.

Additionally, 10 participants (6 women; mean age = 23.7 years, SD = 3.3) took part in the pilot study aimed to compare the pleasantness and intensity of the odors used in the study.

Recruitment was performed through posters and flyers on the campus of Dresden, word of mouth, flyers in local fitness studios, supermarkets or similar. Recruitment was also performed through an advertisement at eBay classifieds.

The data was collected over a period of 12 months (November 2019–November 2020).

### Odors

Table 1 consists of the odors used in the study, together with their characteristics. Odors, including the no-odor placebo condition, were presented through nose clips which allow specific odor presentation to an individual without contaminating the environment. Nose clips were used in both phases of the experiment.

**Table 1:** Odors used in the study.

Number	Name of odor	Description
0	Placebo	No odor
1	Orange	Orange odor
2	“Perfume”	Provided by Takasago citrus; floral; musky; orange
3	l-Laurinal®	Sweety floral—like notes with green citrus and melon undertones

Participants were randomly assigned to one odor group (for orange: 14 women, 13 men, aged from 18 to 39, M = 27, SD = 5.5; for “perfume”: 17 women, 9 men, aged from 20 to 66, M = 32, SD = 12.1; for Laurinal: 11 women, 14 men, aged from 21 to 64, M = 29.7, SD = 11; for placebo: 23 women, 13 men, aged from 19 to 62, M = 28.4, SD = 8). For example, the first participant in the study was allocated in orange group, the second in “perfume”, the third in Laurinal and the fourth in placebo. Odors’ pleasantness was rated after the first 14 days of the experiment on a scale 0–10 (M = 7, SD = 1.9).

### Sleep quality measures

All participants slept at home. They were provided with detailed instructions about all the study aspects (e.g., how to place the nose clip before the night) except for the odors they were presented with. There was no cover story, hence no debriefing.

During the experimental procedure, participants were asked to fill out the sleep diary every morning. The diary included reports on participants’ daytime behavior and sleep perception for all upcoming 14 nights.

Additionally, 66 participants agreed to wear wearable sleep and fitness monitoring devices Fitbit® Charge 2 (Fitbit® charge 2; Fitbit Inc., San Francisco, CA, USA), called further as wSMD, during the whole procedure (28 days). wSMD can be utilized as an alternative measurement of sleep time (i.e., quantity of sleep) and/or sleep efficiency (i.e., quality of sleep), as it allows for measurements of time spent in each of the sleep stages, as well as time spent awake. The wSMD brand family of devices have been shown to have a high intradevice reliability^29^. The participants were asked to synchronize their wSMDs every morning.

### Olfactory, cognitive and non-cognitive testing

**Figure 1:**
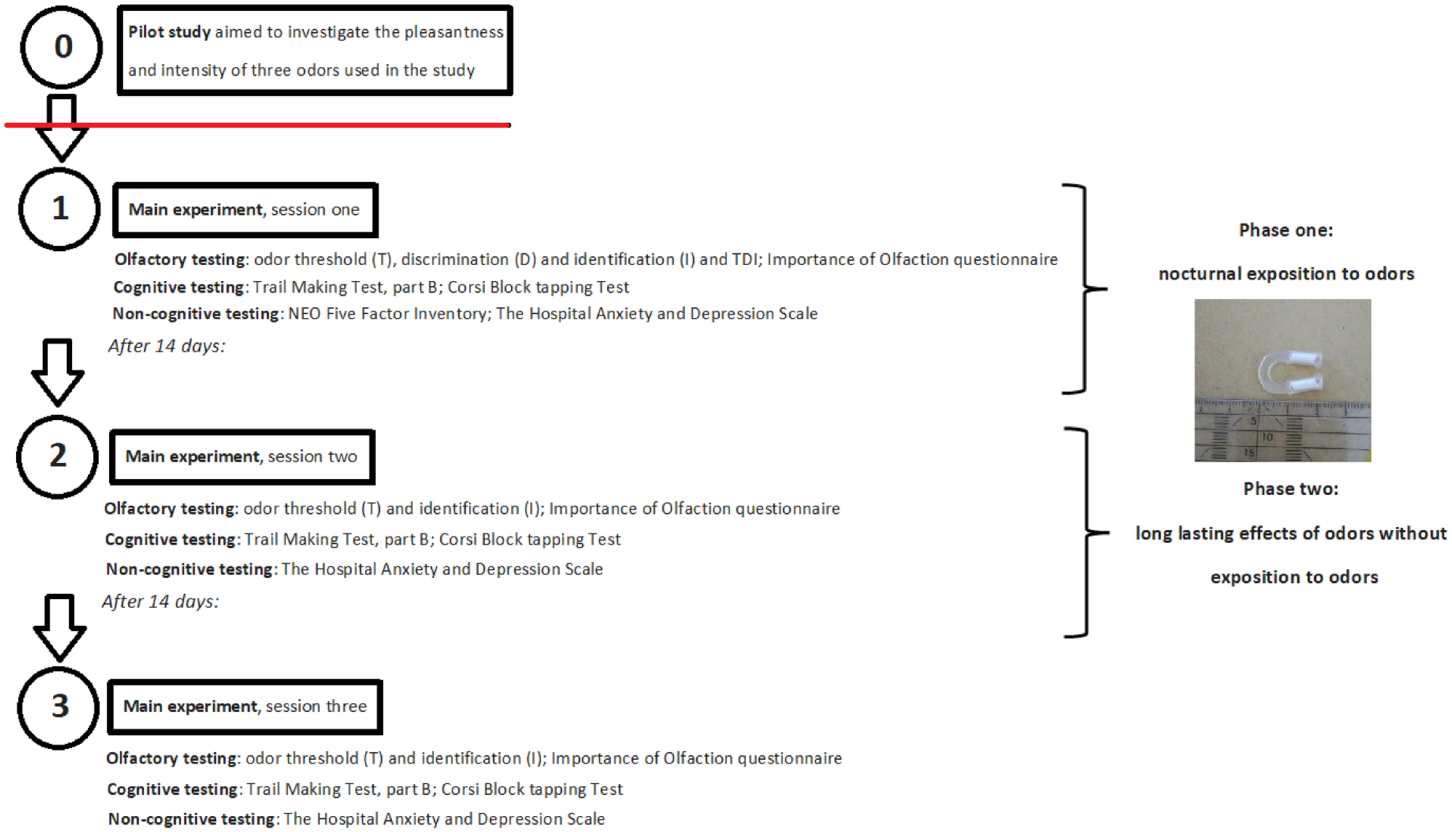
Simplified experimental protocol.

A simplified experimental protocol is presented in Fig. 1. The procedure consisted of three sessions, each separated by 14 days. Descriptive statistics of cognitive and non-cognitive tests are presented in Table 2.

**Table 2:** Descriptive statistics of participants’ performance in four different odor conditions in all the presented tests during the three sessions.

Variable	Odor condition								
	Session	Placebo		Orange		“Perfume”		Laurinal	
		M	SD	M	SD	M	SD	M	SD
**Olfactory testing (Sniffin’Sticks)**									
Odor Threshold	First	7.5	2	6.7	2.3	6.1	2.4	6.1	3.4
	Second	7.3	2.5	6.5	2.5	7	2.7	7.5	4.1
	Third								
Identification	First	14.2	1.1	14.2	1.2	13.8	1.3	13.6	1
	Second	14.6	1.1	13.9	1.2	14.2	1.5	13.9	1.2
	Third								
Discrimination	First	13.7	1.6	13.3	1.5	13.3	1.7	13	1.8
Total assessment of olfactory functions	First	35.4	2.8	34.2	3.6	33.2	3.7	32.6	4.1
Significance of olfaction	First	36.8	7.2	32.3	6.8	34.9	5.9	33.2	5.3
	Second	35.9	6.7	32.8	6.2	34.2	6	31.7	6.2
	Third	35.8	7.1	32.5	6.1	34.1	6.8	32	7.4
**Cognitive testing**									
Executive control (Trail Making Test, part B)	First	50.1	20.9	47.3	10.6	50.3	19.7	57	21.5
	Second	40	16	46.6	29.2	41.8	24.2	43.6	15.6
	Third	36.9	15.4	38.6	17.8	40	21.8	39.3	11.8
Spatial spans (Corsi Block tapping Test)	First	6	1.6	6.5	1.3	6.2	1.6	6.3	1.4
	Second	6.3	1.5	6.3	1.3	6.4	1.6	6.4	1.4
	Third	6.3	1.3	6.6	1.3	6.7	1.6	6.2	1.5
**Non-cognitive tests**									
Anxiety (HADSA)	First	5.1	3.2	5.4	2.6	4.9	3	6.3	2.8
	Second	4.6	3	5.7	3.7	4.7	3.4	6.3	3.2
	Third	5.2	3.7	5.7	2.9	4.2	3.2	6.8	4.1
Depression (HADSD)	First	2.1	2.3	2.9	2.2	2.4	2.7	3.1	2.2
	Second	2.4	2.7	2.8	2	2	2.6	3.8	2.7
	Third	2.3	2.7	2.9	2.1	2	2	4.5	3.6
Personality (NEO Five Factor Inventory)									
Neuroticism	First	17.6	8.1	19	7.7	19	7.5	21.3	7
Extraversion	First	29.3	6	29.4	5.5	29.7	5.6	28.8	5.7
Openness	First	32.5	5.9	30.7	6.3	31.6	6.8	29.3	6.1
Agreeableness	First	32.8	5.7	31.6	5.1	34.1	5	32	6.3
Conscientiousness	First	35.5	5.1	33.7	7.3	34.7	6.7	33.8	6

#### Olfactory function

Olfactory function was assessed in all participants by means of the “Sniffin’ Sticks” test battery (Burghart, Holms, Germany). During the first meeting, odor threshold (T), discrimination (D) and identification (I) were measured, together with the total assessment of olfactory functions (TDI)^30,31^. Only participants who scored 12 or more points in the odor identification test were accepted for the study^31^. This way, five participants (1 woman) were excluded. During the second and the third session, only odor threshold (T) and identification (I) were assessed. These two olfactory factors were chosen because they cover two major domains, threshold and suprathreshold testing. The former is more related to peripheral olfactory functioning, the latter to cognitive functions.

Additionally, the self-assessed significance of olfaction was measured with the Importance of Olfaction questionnaire^32^. The questionnaire consists of 20 items (e.g., ‘without sense of smell, my life would be worthless’) and is a reliable and easy to use instrument.

#### Cognitive testing

Executive control was measured via Trail Making Test, part B (TMT)^33^. TMT is a neuropsychological test that involves visual scanning and working memory. A participant is asked to connect 24 consecutive circles, randomly arranged on a page. The TMT is scored by the time one takes to complete the test. In the case of TMT B the average time to complete this part is 73 s.

Corsi Block tapping Test (CBT)^34^ is a simple way to measure spatial spans. The test was presented to the participants on the computer. Nine blocks positioned randomly on a flat surface were lightening up for a short time while the participant watched. Then the participant was asked to click on the blocks on the screen. In the forward version of the test, the participant repeats the tapping order as presented, while in the backward version, the participant repeats the tapping order in reverse^35^. The test is completed when the participant fails to replicate a given sequence of taps. The most extended series, called the Corsi span, is then marked. The average forward Corsi span for adults ranges between five and seven blocks^34,36^. The CBT is commonly used in neuropsychological and medical studies^35^.

#### Non-cognitive testing

The 60-items NEO Five Factor Inventory (NEO-FFI)^37^ in German adaptation^38^ was presented to the participants to assess their personality. Neo-FFI measures five global dimensions of personality: neuroticism (the predisposition to experience negative affect, such as depression or anxiety), extraversion (the intensity and quantity of social interactions), openness (the predisposition to active searching and appreciation of new experiences), agreeableness (the quality of one’s social interactions along a continuum from compassion to antagonism), and conscientiousness (goal-directed behaviors containing motivation, organization and persistence). Neo-FFI was completed by the participants only in the first session and the results were normal (see Table 2).

The Hospital Anxiety and Depression Scale (HADS)^39^ measures anxiety and depression in a general medical population of patients. It is simple, fast and easy to use^40^. The questionnaire consists of 14 items (7 for anxiety [HADSA], e.g.: I get a sort of frightened feeling like 'butterflies' in the stomach, and 7 for depression [HADSD], e.g.: I have lost interest in my appearance) and takes 2–5 min to complete. The questionnaire's anxiety and depression questions are interspersed, but the final outcome is separated into these two (see Table 2). The questionnaire was presented in German adaptation^41^.

#### A description of a typical experimental session

During the first session, in the beginning, the sense of smell was tested, and then the inclusion and exclusion criteria were checked again. If the tested person fulfilled these criteria, we followed with the information about the study. Afterwards, the participant gave their written consent and was informed that their participation in this study was voluntary and that they could withdraw at any time. This part was followed by a thorough explanation of the sleep diary, including answers to any questions the participant might have asked. Next, explanations of the questionnaires to be filled out afterwards, which represented the second part of each meeting, followed. Each participant could work through the questionnaires peacefully without being observed by the experimenter who left the room for this period. After the experimenter returned, she checked all the questionnaires for completeness. The cognitive tests were then explained and carried out. At the end of the first meeting, the subject was given the clip assigned to her.

The course of the second and third appointments was identical. Firstly, the sense of smell was tested again, with two of the three Sniffin` Sticks tests. Subsequently, new sleep diaries were issued, only for the second session, and the old ones were collected. In addition, at the second meeting, the test person was asked about the pleasantness of the scent and other abnormalities. The subject then filled out the questionnaires he was familiar with, now only two, and completed the cognitive tests, the same as at the first appointment. After three completed meetings, each test person received a moderate expense allowance.

#### Data analyses

##### Pilot study

Pleasantness and intensity of the three odors used in the study were compared by means of repeated measures analysis of variance with (a) rated pleasantness and intensity and (b) orange, Laurinal and “perfume” as within-subject factors.

##### Psychophysical measures

Possible differences in olfactory, cognitive and non-cognitive tests were assessed by means of one-way analyses of variance for repeated measures with odor condition (0, 1, 2 or 3) as between-subject factor. Here, we also investigated the impact of perceived odor pleasantness on the declared sleep quality. Following the ratings of all delivered odors’ pleasantness, participants were divided into two groups: (a) assessing the odors as less pleasant (≤ 7; 55 participants; 51.8%); (b) assessing the odors as more pleasant (≥ 8; 59 participants; 48.2%). Obtained odor pleasantness rating was used as between-subjects factor. For these analyses, the placebo group was excluded.

##### Sleep diary

Analyses were conducted separately for each individual question for the first, seventh, thirteenth night (phase 1), fifteenth, twenty-first, and twenty-eight night (phase 2), employing Repeated Measures Anova. Thus, eight separate models were constructed with nights (1, 7, 13), and phases (1 vs 2) as within-subjects factors and odor condition (0,1,2 or 3) together with odors pleasantness rating as between subject factors. We did not investigate the question concerning yesterday’s physical activity because there were about 20% missing data. Scoring for the sleep diary is described in supplementary Table 1. For the purpose of the analyses, the scale has been changed in ways that the lowest numbers respond to the lowest score and the highest to the highest number. For example, regarding the item ‘how many hours did you sleep’ 0 responds to 0–4 h and 4 to 10–12 h. To control for multiple comparisons, Bonferroni corrections were applied where appropriate.

##### wSMD measures

Data were analyzed both individually^42^ and grouped in the following way:

- sum of minutes asleep; time spent in bed; minutes REM sleep, and minutes of deep sleep (N3) were summed up into a category of ‘positive’ sleep quality;
- sum of minutes awake andminutes of light sleep (N2) were summed up into a category of ‘negative’ sleep quality.

Both ‘positive’ and ‘negative’ sleep quality of consecutive two nights of each phase were combined into one. This way, we obtained seven nights for each phase.

Possible differences in the sleep quality, as measured via wSMD, were assessed by one-way analyses of variance for repeated measures, separately for ‘positive’ and ‘negative’ sleep quality. Within-subject factor included nights and phases (1, 2), with odor condition (0, 1, 2 or 3) and odors pleasantness rating as between-subject factors. In order to control for multiple comparisons, Bonferroni corrections were applied where appropriate.

Additionally, we further examined the effect of odor condition on sleep quality with Bayesian statistics^43^. The Bayes Factor (B) is a method that weighs evidence and shows which of two hypotheses is better supported and to what extent. Adopting the B in statistical inference, it can be examined whether the data provided robust support for the null hypothesis, the alternative hypothesis, or whether the analysis is inconclusive and more data need to be collected to provide clear evidence^44^. Furthermore, Bayesian statistics are resistant to multiple comparisons.

Data are presented as mean values (± standard deviation). Statistical analyses were performed using JASP v. 0.11.2 (www.jasp-stats.org), with p < 0.05 set as the level of significance.

## Results

### Pilot study: comparison of the pleasantness and intensity of the three odors

**Figure 2:**
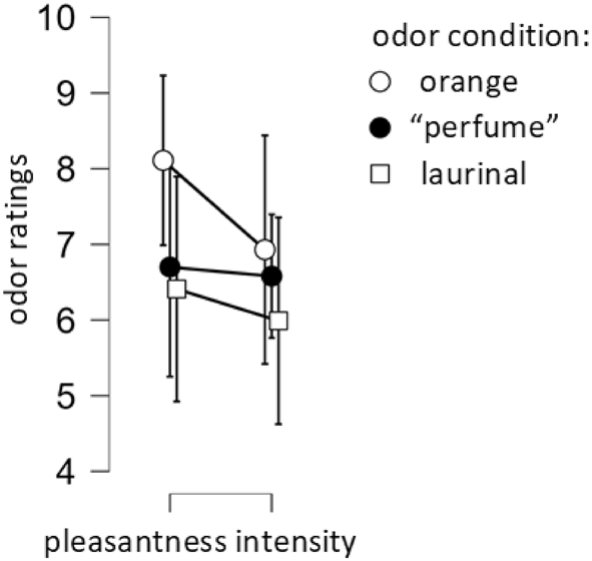
Pleasantness and intensity ratings of three odors used in the study—the higher the scores the more intense the odors. Pleasantness ratings of 0 indicate very unpleasant perceptions, ratings of 10 very pleasant sensations, scores of 5 indicate a hedonically neutral perception of the odor.

No interaction was noticed between either intensity or pleasantness and three odors used in the study (F[2,18] = 0.6; η^2^ = 0.02; p = 0.57) (Fig. 2).

### Analyses of psychophysical measure

#### Non-cognitive testing

**Figure 3:**
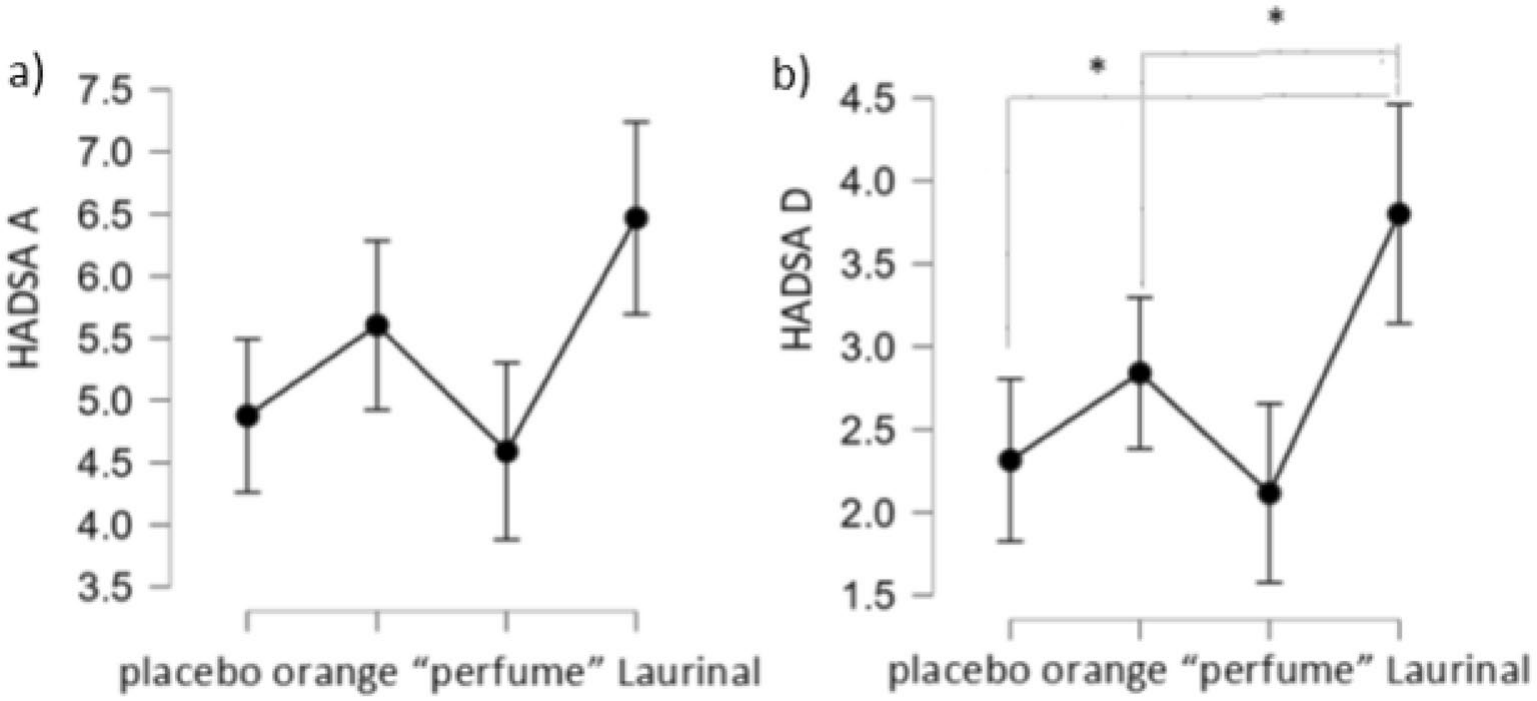
(**a**, **b**) (from the left). HADSA and HADSD scores in different odor conditions. Note different Y-axis.

HADSA scores’ analyses showed between-subjects effect for odor condition (F[3,105] = 2.9, p = 0.04). Specifically, participants in the Laurinal group tended to score higher—thus, reported higher anxiety level—compared to those in the “perfume” group (p = 0.052, Bonferroni corrected) (Fig. 3a).

The same effect was even more visible in case of HADSD scores (F[3,105] = 3.6, p = 0.016), with participants in the Laurinal group reporting higher depression severity compared to those in “perfume” and placebo groups (for “perfume”: p = 0.035; for placebo: p = 0.027, all p’s Bonferroni corrected) (Fig. 3b).

Bayes factor revealed that in case of both anxiety and depression, the null model was indistinguishable from the model with the main effect of odor (for anxiety: B01 = 0.8; for depression: B01 = 0.3).

#### Olfactory testing

**Figure 4:**
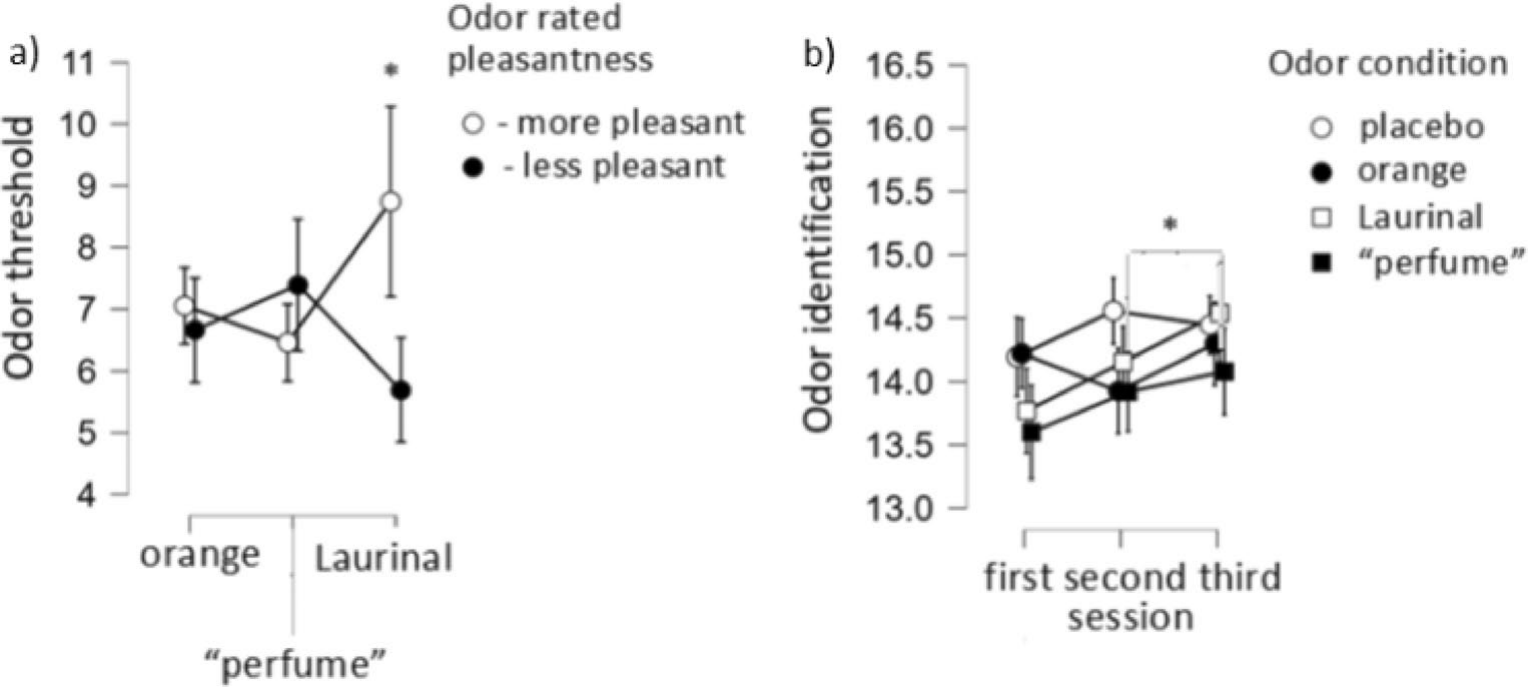
(**a**, **b**) Odor threshold in the Laurinal group for participants who rated the odors as more and less pleasant, respectively; Odor identification in three sessions for different odor conditions. Note different Y-axis.

Increase in odor threshold was noticed (F[2,212] = 12.4, p < 0.001) together with between-subjects effects regarding the interaction between odor pleasantness rating and odor condition (F[2,72] = 4.3, p = 0.017). Specifically, odor threshold performance was higher in the group presented with Laurinal, for participants who rated the odors as more pleasant than those who rated them as less pleasant (p = 0.035, Bonferroni corrected) (Fig. 4a).

Alike, increase in odor identification was demonstrated (F[2,212] = 9.5, p < 0.001). Here, at the second measurement participants exhibited an improved odor identification function (F[6,212] = 2.3, p = 0.039). Specifically, participants in the “perfume” group had lower scores between the second and third measure (p = 0.003, Bonferroni corrected) (Fig. 4b).

Bayes factor revealed that odor condition did not affect odor threshold (B01 = 5) while for identification performance. the null model was indistinguishable from the model with the main effect of odor (B01 = 3.6).

#### Cognitive testing

TMT scores analyses showed a decrease in scoring (F[1.8,202] = 27.9, p < 0.001, Greenhouse–Geisser correction) while Corsi test scores analyses demonstrated a tendency of increase (F[1.8,190.4] = 3.2, p = 0.05, Greenhouse–Geisser correction). Alike, an increase in total correct trials (F[1.9,197,9] = 4.9, p = 0.01, Greenhouse–Geisser correction) and memory span (F[1.9,197.8] = 4.5, p = 0.015, Greenhouse–Geisser correction) was noted.

Bayes factor indicated that odor condition did not influence either TMT scores (B01 = 9.3) nor corsi test outcome (for blockspan: B01 = 9.5; for total correct trials: B01 = 7.5; for total score: B01 = 7.4; for memory span: B01 = 7.5). In other words, the null hypotheses concerning the lack of relationship between these variables was, correspondingly, 9 and 7 times more probable than the opposite assumption.

#### Analyses of the sleep quality measures obtained via sleep diary

Regarding the first question, “how fast did you fall asleep”, participants declared faster falling asleep in the second phase compared to the first one (F[1,106] = 6.1, p = 0.016). Furthermore, at the 15th night (second phase), participants reported faster falling asleep than at the first night (first phase) (F[2,212] = 4.2, p = 0.016; p = 0.003, Bonferroni corrected).

Regarding the second question, “did you wake up”, participants declared more awakenings in the second phase compared to the first one (F[1,106] = 5.6, p = 0.019). Furthermore, main effect of nights (F[2,212] = 10.3, p < 0.001) and an interaction between nights and phases (F[2,212] = 2.5, p = 0.086) were demonstrated, however the latter on a trend level only. Specifically, in nigh one (first phase), participants declared fewer awakenings compared to the following nights: 13th (first phase), 21st (phase 2), 28th (phase 2) (all p’s < 0.001, all p’s Bonferroni corrected), and night 15th (phase 2), however here the effect was noticed on a trend level only (p = 0.052, Bonferroni corrected).

Regarding the fourth question, “how was your sleep”, participants declared higher sleep quality in some of the nights of the second phase compared to the first one (F[2,212] = 4.4, p = 0.014). Post-hoc tests revealed that higher sleep quality was reported on night 13th (first phase) (p = 0.009, Bonferroni corrected) and fifteen (second phase), however here, the effect was noted on a trend level only (p = 0.06, Bonferroni corrected).

Regarding the sixth question: “how well-rested did you get up”, participants reported more feeling of being rested in the second phase compared to the first one (F[2,212] = 4.5, p = 0.012). Specifically, the reported feeling of being rested increased gradually: both on nights thirteen (first phase) and twenty-eight (second phase), sleep quality was rated higher compared to the first night (phase 1) (for the 13th night: p < 0.001, for the 27th night: p = 0.012, all Bonferroni corrected). Also, on night seven (first phase), participants tended to declare higher sleep quality compared to night one (first phase) (p = 0.052, Bonferroni corrected).

**Figure 5:**
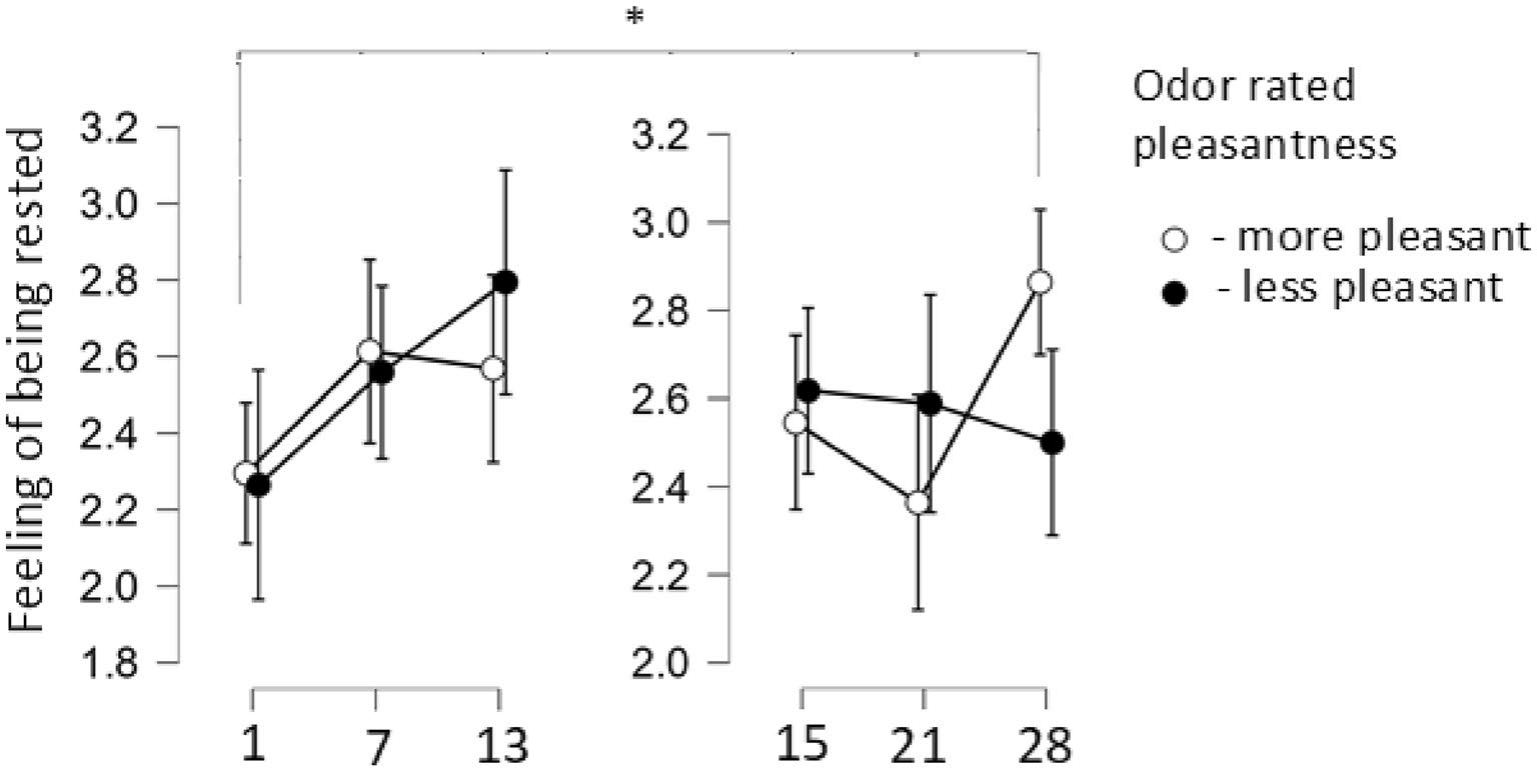
Feeling of being rested after getting up as assessed by participants who rated the odors pleasantness.

Furthermore, main effect of nights, phases, and odors pleasantness rating was demonstrated (F[2,144] = 4.6, p = 0.012). Participants who rated the odors as more pleasant reported more feeling of being rested on night twenty-eight (second phase) compared to night one (first phase) (p = 0.014, Bonferroni corrected). In turn, those who rated the odors as less pleasant, tended to declare more feeling of being rested on night thirteen (p = 0.014, Bonferroni corrected) compared to night twenty-eight (second phase) (Fig. 5).

As indicated by Bayes factor, in neither of the above questions odor condition influenced sleep quality ratings (for the first question: B01 = 18.4; for the second question: B01 = 19.1; for the third question: B01 = 25; for the fourth question: B01 = 34.3; for the fifth question: B01 = 5.6; for the sixth question: B01 = 8.2; for the seventh question: B01 = 18.8; for the eight question: B01 = 9). In other words, the null hypotheses concerning the lack of relationship between these variables was at least 5—and maximally 34 times more probable than the opposite assumption.

### Analyses of sleep quality measures obtained via wSMD

Descriptive characteristics of the aspects of sleep quality obtained via wSMD for each phase are presented in the supplementary Table 2.

Considering individual categories, for ‘minutes awake’ general increase in time spent awake was noticed (F[6,288] = 2.9, p = 0.009) between night 1 and 14 (p = 0.006, Bonferroni corrected). Furthermore, time spent in bed increased in the second phase (F[1,288] = 4.9, p = 0.032).

Bayes factor indicated that odor condition did not affect sleep quality in case of ‘minutes asleep’: B01 = 5.3. As regards other categories, the null model was indistinguishable from the model with the main effect of odor (for ‘minutes awake’: B01 = 1.9; for ‘number of awakenings: B01 = 2.7; for ‘time in bed’: B01 = 5.5; for ‘rem sleep’: B01 = 1.3; for ‘deep sleep’: B01 = 1.9; for ‘light sleep’: B01 = 3.2).

Regarding summed categories of sleep quality, we found that ‘positive’ sleep quality increased; in the second phase; i.e. sleep quality improved (F[1,288] = 5.5, p = 0.024). Here, theBayes factor showed that odor condition did not influence this aspect (B01 = 12.6). As for the negative sleep quality, the null model was indistinguishable from the model with the main effect of odor (B01 = 2.9).

## Discussion

We systematically examined the idea of whether Laurinal, orange odor, and “perfume” in comparison to a no-odor condition, influence sleep quality of a large group of healthy participants. Specifically, we aimed to investigate the effects of continuous odor exposure during sleep on physical parameters, and subjective reports collected via a sleep diary. In contrast to the majority of previous studies^4–7,23^; but see:^28^, the present results indicate that odor condition has no consistent or significant effect on sleep which was confirmed by Bayesian anlayses.

One plausible explanation of the lack of odors’ impact on sleep quality might be the experimental conditions employed in the present study. Nose clips used to deliver odors do not mimic ambient atmosphere in which odor exposure takes place at people’s homes. While fresh and pleasant bedroom odors receive a central role in sleep hygiene^45^, typical ways to obtain this goal are airing the room, using scented candles, incense or adding scents (e.g., lavender) to linens. A 19th-century anecdotal report regarding the impact of odors on sleep quality refers to dropping perfumes on the pillow [cited from^46^]. Compared to the use of nose clips these techniques appear to be more convenient. In addition, they also allow to share the odors with others, such as romantic partners, therefore providing essential social context to the odor exposition. In turn, social meaning given to the odors can enhance sleep quality^47^; see also:^48^.

Additionally, in the present study, individual odors differed very slightly in influencing affective or psychophysical states of participants. Specifically, between-subject analyses revealed that after the use of “perfume”/placebo, participants reported lower depression severity compared to the use of Laurinal. Furthermore, again on between-subjects effects level, participants in the “perfume” group tended to report lower anxiety level compared to those in the Laurinal group. Besides, odor threshold performance was higher in the group presented with Laurinal, for participants who rated the odors as more pleasant compared to those who rated them as less pleasant, as shown by between-subject analyses. An open question remains why some of the odors employed in the study caused such effects, and some did not.

Odors are well-established to impact physiology and mood (see for review:^49^), leading to decrease in anxiety and a greater sense of calmness, relaxation, and contentment^3,50–54^. Why results from the present, rigorous study indicate only spurious effects of odors in this area remains an open question. However, it must be noted that a very limited number of odors has been associated with a consistent impact on mood, whilst exposure to others has produced varied or contradictory results^53^. For example, while exposure to orange oil has been reported to improve mood and reduce anxiety^3,51^, olfactory stimulation with rosemary has been observed to either increase anxiety^54^ or decrease it^50^.

These possibly more selective abilities of odors might be explained, on one hand, by chemosignals in some essential oils that, following absorption in the respiratory tract, may cause sedative and anxiolytic effects through specific effects in the central nervous system^55–58^. On the other hand, odors tend to readily acquire the affective valence of the situation in which they were experienced via evaluative conditioning^59^, and hence become liked or disliked^60^. Going further, odors that are contextually linked with positive experiences can also exert an anxiolytic effect, perhaps through a reduction in sympathetic tone^48^. Villemure and colleagues^61^ showed that when presented to a self-selected pleasant odor, participants reported mood improvement and decrease in anxiety and perceived pain unpleasantness. On the contrary, exposure to a smell that the participants themselves picked as unpleasant resulted in the opposite. Thus, the impact of odors on affective and psychophysical states appears a complex issue with a number of factors such as biological components, unique contextual background and perceived hedonics as potential mediators.

Interestingly, the present results indicate that except for real odor perception, also imaginary sniffing might contribute to improvement in affective state. Exposition to placebo odor resulted in decrease of depression severity, what can be explained by presumably positive expectations of participants regarding the odors’ effects. Masaoka et al.^62^ demonstrated that blind exposition to no odor, when associated with certain positive expectations about odor’s effects, might bring beneficial results. This effect can be caused by breathing patterns that change while imaginary sniffing: analogous to real odor perception, imagination of a pleasant odor is accompanied by a larger sniff^63–65^.

Present results indicate that perceived odor’s pleasantness enhanced some of the positive assessment, which is in line with previous studies^66–72^. Regarding individual questions of the sleep diary, participants who rated the odors as more pleasant tended to report an increase in feeling of being rested during both phases, especially when comparing the end of the experiment with the beginning. Interestingly, this effect was not present in those who rated the odors as less pleasant—instead, they tended to declare less feeling of being rested at the end of the second phase compared with the end of the first phase. Some of those effects have been noted in previous studies^7,23,73^. For example, Goel and Lao^73^ demonstrated that peppermint, perceived as pleasant, decreased a feeling of fatigue. Exposure to pleasant odors was also shown to be associated with positive dream contents^7^. Lastly, participants who rated the odors as unpleasant might be slightly anhedonic compared to the others which results in diverse odor encoding^74^.

Affective perception evoked by an odor is crucial for olfactory processing^75^. Already at the level of sniff response pleasant odors drive stronger sniffs and unpleasant odors drive weaker sniffs^63,76^. Anderson et al.^77^ found that the pleasantness of an odor was processed in spatially separate areas of the orbitofrontal cortex (OFC), which play a significant role in emotional processing. Present results emphasize the idea that the subjective perception of an odor’s hedonic value might be crucial for sleep quality, not the odors themselves.

One would expect that overnight odor exposure would increase or decrease sleep quality and psychophysical measures consistently during the first phase and then return to baseline or stabilize in the second phase. While some results fit into this hypothesis, others do not. For example, odor identification kept increasing from sessions 2 to 3. Regarding sleep diary, participants reported more feeling of being rested in the second phase compared to the first one. Also, “positive” sleep quality measured with wSMD was higher in the second phase compared to the first one. One plausible explanation might be the indirect effects of frequent odor exposition, such as increased attention to odors in case of odor identification^78^. Furthermore, mood improvement, here presumably reflected in a feeling of being more rested, was demonstrated to accompany olfactory performance amelioration^79^.

To conclude, the present results show that odor condition had no consistent or significant effect on sleep in healthy participants. It can be possibly explained by exposure to odors via nose clips that may not mimic an ambient atmosphere in which odor exposure is usually employed at people’s homes. Furthermore, only spurious effects of individual odors on affective or psychophysical states of participants were noticed. In turn, the present data support the view that subjectively perceived pleasant odors enhance the positive assessment of sleep quality. Overall, the present results indicate that the subjective perception of an odor’s hedonic value is crucial for sleep quality, not the odors themselves.

## Supplementary Information

Supplementary Information.

## Data Availability

The datasets analyzed during the current study are not publicly available due to the privacy of the participants but are available from the corresponding author on reasonable request.
