# Supplementary material for: Effects of odors on sleep quality in 139 healthy participants
Source: Sci Rep. 2022 Oct 13;12:17165. doi: 10.1038/s41598-022-21371-5 (PMC9562345; doi:10.1038/s41598-022-21371-5)
Supplement: Supplementary file 1 — Supplementary Information. [file 41598_2022_21371_MOESM1_ESM.docx]

Supplementary Materials

Table 1

Sleep diary

| date |  | | | | | | | |
| --- | --- | --- | --- | --- | --- | --- | --- | --- |
|  | When did you fall asleep? When did you wake up? | | | | | | | |
|  | fall asleep at: woke up at: | | | | | | | |
|  | How fast did you fall asleep? | | | | | | | |
|  | ☐**0**  I don´t know | ☐**2**  right away | | ☐**1**  after a short amount of time | ☐**-1**  after some time | | ☐**-2**  not for a long time | |
|  | Did you wake up? | | | | | | | |
|  | ☐**0**  I don´t know | ☐**2**  no | | ☐**1**  once | ☐ **-1**  multiple times | | ☐ **-2**  constantly | |
|  | Did you get up? | | | | | | | |
|  | ☐**0**  I don´t know | ☐**2**  no | | ☐**1**  once | ☐ **-1**  multiple times | | ☐ **-2**  constantly | |
|  | How was your sleep? | | | | | | | |
|  | ☐**2**  excellent | ☐**1**  good | | ☐**0**  not so good | ☐**-1**  bad | | ☐ **-2**  very bad | |
|  | Were you dreaming? | | | | | | | |
|  | ☐**0**  I don´t know | ☐**1**  yes, something | | ☐ **2**  yes, very pleasantly | ☐**-1**  yes, not very pleasantly | | ☐**-2**  yes, it was a nightmare | |
|  | How well rested did you get up? | | | | | | | |
|  | ☐ **2**  very well | ☐ **1**  well | | ☐**0**  quite | ☐ **-1**  not well | | ☐ **-2**  I feel exhausted | |
|  | How many hours did you sleep? | | | | | | | |
|  | **-2**  0-4 | **0**  4-6 | | **2**  6-8 | **1**  8-10 | | **-1**  10-12 | |
|  | Did you take a nap yesterday? | | | | | | | |
|  | **0**  no | **2**  < 0,5 hour | | **1**  0,5-1 hour | **-1**  1-2 hours | | **-2**  > 2 hours | |
|  | yesterday´s physical activity | | | | | | | |
|  | **-2**  much less | **-1**  less | **0**  NORMAL | | | **1**  more | | **2**  much more |

Supplementary Table 2

Mean values ±SD, Min. and Max. of the sleep quality measures obtained via wSMD for each phase separately

|  | M | SD | Min. | Max. |
| --- | --- | --- | --- | --- |
| **minutes asleep – phase 1** | 5372.203 | 853.125 | 1844 | 6927 |
| **minutes asleep – phase 2** | 5466.763 | 775.073 | 2344 | 7452 |
| **minutes awake – phase 1** | 661.644 | 205.54 | 117 | 1048 |
| **minutes awake – phase 2** | 696.441 | 184.147 | 225 | 1286 |
| **number of awakenings – phase 1** | 280.034 | 136.345 | 34 | 573 |
| **number of awakenings – phase 2** | 309.983 | 125.144 | 25 | 590 |
| **time in bed in minutes – phase 1** | 6354.017 | 748.504 | 4165 | 8018 |
| **time in bed in minutes – phase 2** | 6332.458 | 866.804 | 2747 | 8738 |
| **minutes REM sleep – phase 1** | 856.237 | 410.893 | 48 | 1688 |
| **minutes REM sleep – phase 2** | 933.475 | 381.831 | 128 | 1667 |
| **minutes light sleep – phase 1** | 2359.186 | 1119.512 | 236 | 4507 |
| **minutes light sleep – phase 2** | 2575.644 | 976.677 | 206 | 4680 |
| **minutes deep sleep – phase 1** | 677.881 | 330.287 | 46 | 1544 |
| **minutes deep sleep – phase 2** | 761.169 | 288.583 | 76 | 1301 |
